# Psychological flexibility as a mediator between social participation and retirement adaptation: an ACT intervention study from the perspective of active aging

**DOI:** 10.3389/fpubh.2026.1859171

**Published:** 2026-06-29

**Authors:** Fang Yang, Xiaohua Shen, Xiaolan Zhang, Jian Liu

**Affiliations:** 1 School of Mental Health and Psychological Science, Anhui Medical University, Hefei, China; 2 Department of Teaching, Seventh People’s Hospital of Hangzhou, Hangzhou, China; 3 Department of Nursing, Hangzhou Wuyunshan Hospital (Hangzhou Health Promotion Research Institute), Hangzhou, China; 4 Department of Employee Health Promotion, Hangzhou Wuyunshan Hospital (Hangzhou Health Promotion Research Institute), Hangzhou, China

**Keywords:** acceptance and commitment therapy, older adults, intervention, positive aging, psychological flexibility, retirement adaptation

## Abstract

**Introduction:**

This study aims to examine the effectiveness and mechanisms of Acceptance and Commitment Therapy (ACT) in promoting retirement adaptation among older adults, and to analyze the mediating role of psychological flexibility within the framework of positive aging and successful aging theory.

**Methods:**

A cross-sectional survey of 145 participants was conducted to establish a mediation model exploring psychological flexibility between social participation and retirement adaptation. Subsequently, 77 eligible participants were randomly assigned to an ACT intervention group (*n* = 40) or a control group (*n* = 37). The intervention consisted of six weekly 40-min sessions. Both groups were assessed on retirement adaptation and psychological flexibility before and after the intervention. Mediation analysis controlled for demographic variables, and effect sizes were reported alongside *p*-values.

**Results:**

Psychological flexibility partially mediated the association between social participation and retirement adaptation (indirect effect = 0.189, 95% CI [0.098, 0.298]), accounting for 29.73% of the total effect. ACT intervention significantly improved both retirement adaptation and psychological flexibility (*p <* 0.001), with more pronounced improvements in the dimensions of retirement satisfaction and work nostalgia.

**Discussion:**

ACT may promote retirement adaptation by enhancing psychological flexibility, providing preliminary evidence for psychological interventions under the active aging framework. Causal inferences from the cross-sectional mediation model require validation in longitudinal designs.

## 1 Introduction

Retirement is a significant turning point in later life, marking not only the end of one’s professional career but also a major shift in personal social roles and daily life patterns. This transition is often accompanied by a decrease in income, reduced social interactions, a restructuring of daily life, and psychological role conflicts, which increase the risk of psychological adaptation difficulties and the onset of mental health issues such as depression and anxiety (1–3). Poor retirement adaptation can lead to a series of psychological disorders and psychosomatic dysfunction, sometimes referred to as “retirement syndrome” (4). Many older adults face psychological distress after retirement, including feelings of loneliness, loss, and anxiety. Research indicates that poor adaptation to retirement is associated with depressive symptoms, with the prevalence of depression among individuals aged 60 and above in China reaching 53.02% (5). As the global aging process accelerates, effectively promoting positive retirement adaptation in older adults has become a critical issue in gerontology and public health.

The concept of retirement adaptation is not yet universally defined, with scholars offering various perspectives. In general, retirement adaptation is considered a multidimensional process that involves how individuals cope with physiological, psychological, social, and emotional changes brought about by retirement (4, 6, 7). Existing studies suggest that social participation is a core protective factor for retirement adaptation, as it helps mitigate the risk of identity loss through social support, role restructuring, and a sense of life meaning (8). Among older adults, social participation is not only directly linked to life satisfaction but can also enhance psychological resources, such as self-efficacy, thereby facilitating better adaptation (9). A structural equation model by Davies et al. (10) shows that social participation can indirectly improve mental health by enhancing cognitive flexibility, which in turn strengthens the positive effects of social participation (11). Thus, we hypothesize that:

*H1*: Social participation significantly predicts retirement adaptation.

*H2*: Psychological flexibility may mediate the relationship between social participation and retirement adjustment.

Cassanet et al. (12) pointed out that retirement adaptation varies among individuals, with some needing psychological health support during this transition. Psychosocial interventions have proven effective in improving retirement well-being in older adults (12). In recent years, Acceptance and Commitment Therapy (ACT), centered around the concept of psychological flexibility, has gained attention in geriatric populations. Psychological flexibility refers to the ability to maintain an open attitude toward internal experiences (such as emotions and thoughts), consciously engage in the present moment, and take effective action in line with personal values. This ability plays a key mediating role in the relationship between social participation and retirement adaptation, and is considered an important psychological resource for promoting mental health and successful aging (13). Within the active aging framework, it helps individuals navigate life transitions and sustain social engagement (14). ACT, as a third-generation cognitive behavioral therapy, is rooted in functional contextualism and relational frame theory. It emphasizes developing psychological flexibility through six core processes: acceptance, cognitive defusion, present-moment contact, self-as-context, values clarification, and committed action, rather than attempting to eliminate or control negative internal experiences (15). Hayes and Pierson have applied ACT to help older adults cope with post-retirement emotional and psychological changes, focusing on reducing experiential avoidance and cognitive fusion to reconnect with personal values and meaning. Furthermore, Karlin et al. (16) showed ACT effectively alleviates depression and anxiety in older adults and is better suited to their psychological characteristics than traditional cognitive-behavioral therapy (17). However, few empirical studies have examined ACT’s causal effects on retirement adaptation or its mechanisms. In particular, the mediating role of psychological flexibility between social participation and retirement adaptation, and ACT’s influence on specific dimensions such as retirement satisfaction and work nostalgia, remain underexplored. Existing research mostly addresses symptom reduction or general mental health, leaving a gap in studies linking behavior, psychological resources, and adaptation outcomes within retirement transitions, especially under active and successful aging frameworks. Therefore, this study further proposes:

*H3*: ACT intervention effectively improves retirement adaptation levels in older adults.

This study investigates the effectiveness and underlying mechanisms of ACT in promoting retirement adaptation among older adults using a mixed-methods design. First, a cross-sectional survey tested a mediation model of psychological flexibility linking social participation to retirement adaptation, providing a theoretical foundation for subsequent interventions. Second, a randomized controlled trial examined ACT’s causal effects on retirement adaptation and psychological flexibility and clarify the mechanisms by which ACT exerts these effects. Unlike previous studies, this research integrates two complementary frameworks: active aging, which emphasizes optimizing health, participation, and functional maintenance, and successful aging, which focuses on positive outcomes across physiological, psychological, and social domains. In this model, social participation represents the behavioral pathway, psychological flexibility the core psychological resource, and retirement adaptation the key developmental outcome. This integrated “behavior–psychological resources–adaptation outcomes” framework advances understanding of the psychological mechanisms underlying retirement adaptation and provides empirical support for theory integration. By validating ACT’s intervention mechanism, the study offers evidence-based guidance for strategies targeting psychological flexibility, with important theoretical and practical implications for enhancing older adults’ quality of life and promoting active aging (18).

## 2 Participants and methods

### 2.1 Participants

This study comprised two independent sub-studies. Study 1 was conducted between April and May 2024 in Hangzhou, Zhejiang Province, China. A cross-sectional survey was administered to 145 individuals in community streets, health centers, and nursing homes. Study 2 was conducted with 80 retired cadres selected from the Wuyunshan Cadre Sanatorium in Hangzhou. Inclusion criteria were: (1) Females aged 50 years and older and males aged 60 years and older, to align with the average retirement age. All participants were under 80 years of age. (2) Normal vision or corrected vision, right-handed, to ensure consistency for potential future neurophysiological measurements. (3) No cognitive or mental disorders. (4) Retired. (5) Agreement from the patient and their family to participate in the study. Exclusion criteria were: (1) Cognitive impairment or mental disorders, excluded based on the Mini-Mental State Examination. (2) Severe physical diseases, paralysis, bedridden individuals, etc. (3) Re-employed retirees. Of the 80 initially recruited, 3 withdrew before randomization due to scheduling conflicts and were excluded from analysis. The remaining 77 participants completed randomization, baseline assessment, and post-intervention assessment (attrition rate = 0%). A total of 77 participants were included in the study after excluding those who withdrew, with 40 assigned to the ACT intervention group and 37 to the control group. Randomization was conducted by an independent statistician using a 1:1 sequence generated from a random number table. Allocation was concealed in sequentially numbered, opaque, sealed envelopes, which were opened by a research assistant not involved in outcome assessment after baseline testing.

### 2.2 Research instruments

#### 2.2.1 Mini-mental state examination

A commonly used tool consisting of various questions covering aspects such as temporal orientation, spatial orientation, immediate memory, attention and calculation, delayed memory, language function, and visuospatial ability (19).

#### 2.2.2 Demographic questionnaire

This questionnaire includes information on gender, age, years of education, marital and reproductive status, retirement time, and family history of mental illness in older adults participants.

#### 2.2.3 Acceptance and action questionnaire-II (AAQ-II)

Used to assess an individual’s degree of experiential avoidance and psychological rigidity, it is one of the most widely used tools for measuring psychological flexibility. The AAQ-II includes 7 items scored on a 7-point scale (1 = never true to 7 = always true), with higher scores indicating higher levels of experiential avoidance and psychological rigidity, and lower levels of psychological flexibility. The AAQ-II has demonstrated good reliability and high structural, convergent, and discriminant validity in various studies.

#### 2.2.4 Retirement adjustment inventory

This Chinese version of the Retirement Adjustment Questionnaire was translated by Zhang and Wang (20). It includes 13 items that measure three dimensions: retirement satisfaction, retirement loss, and nostalgia for work. The scale uses a 5-point Likert scale and assesses the overall adaptation level of retirees post-retirement. The scale has high reliability and validity, and can comprehensively reflect “retirees’ psychological and social adaptation status” (20).

### 2.3 Study design

Study 1 used a convenience sampling method to collect data offline in community health centers, nursing homes, and sanatoriums in Hangzhou, Zhejiang Province, with a total of 145 participants. Study 2 employed a pre- and post-test experimental design with an intervention group and a control group to examine the impact of psychological flexibility on retirement adaptation and social participation among older adults.

Pre-test: Before the intervention, all participants provided informed consent, and baseline assessments were conducted for both the intervention and control groups.

Intervention: Based on the guidance of ACT theory and research on social participation, an intervention plan was developed and implemented. The intervention included weekly group counseling sessions focused on ACT, lasting 6 weeks, with each session lasting 40 min, and delivered by licensed clinical psychologists with at least 5 years of experience in older adult mental health. The control group participated in older adults health education courses during the intervention period for comparison.

Post-test: After the final group counseling session, assessments were repeated in the same manner as the pre-test (Tables 1, 2).

**Table 1:** Control group courses.

Course title	Course content
Theme 1: Health Group Counseling – “Sleep Disorders Program”	Introduction to sleep measurement methods, characteristics of sleep in older adults, the importance of sleep, sleep assessment techniques, and strategies for improving sleep quality.
Theme 2: Health Lecture – “Health: From the Perspective of Successful Aging”	Discussion of health from multiple perspectives, including body shape, organ function, disease resistance, and environmental adaptability; introduction of new health concepts and the significance of successful aging.
Theme 3: Health Lecture – “Carrying the Load: Body Weight and Cardiopulmonary Resuscitation”	Explanation of how older adults can assess their body shape and nutritional status, current medical technologies, the risks associated with obesity in older adults, and strategies for weight management.
Theme 4: Psychological Group Counseling – “Embracing Happiness and Well-being”	Group-based emotional activities designed to promote psychological well-being, including collaborative handicraft activities.
Theme 5: Health Lecture – “The Importance of Vitamin D”	Overview of trace elements and their functions, with a focus on the importance of vitamin D for older adults.
Theme 6: Summary and Social Activity	Organization of singing and dancing performances, along with a review and reflection on the enjoyable experiences throughout the program.

**Table 2:** Intervention group courses.

Course title	Course content
Session 1: Mindfulness and Self-Observation	Introduction to the concept of mindfulness, which involves observing one’s thoughts, emotions, and bodily sensations in the present moment without judgment. Through exercises and discussions, older adults are guided to cultivate mindfulness and self-observation skills to better understand and manage their inner experiences.
Session 2: Acceptance and Emotional Regulation	Explanation of the concept of acceptance, which involves acknowledging and embracing one’s emotions rather than avoiding or resisting them. Through exercises and discussions, participants are encouraged to accept their emotions, thereby reducing psychological stress and anxiety.
Session 3: Awareness and Cognitive Restructuring	Introduction to cognitive defusion—the ability to observe thoughts without being dominated by them, reducing the impact of rigid beliefs such as “retirement equals worthlessness.” Through mindfulness exercises and metaphor techniques (e.g., “leaves on a stream”), older adults learn to establish a flexible relationship with negative thoughts and contact present-moment experiences with openness and non-judgment.
Session 4: Values Clarification and Behavioral Choices	Guiding older adults to clarify their core values and goals, i.e., what they truly want to achieve in life. Through discussions and exercises, participants learn to make behavioral choices aligned with their values, promoting personal growth and a sense of fulfillment.
Session 5: Behavioral Activation and Commitment	Emphasizing the importance of behavior in achieving one’s values and goals. Exercises and discussions help older adults create specific action plans and encourage them to take positive actions to fulfill their commitments.
Session 6: Integration and Ongoing Growth	A review of the content from the previous five sessions, helping older adults integrate the knowledge and skills learned. Participants are encouraged to maintain their abilities in mindfulness, acceptance, awareness, and self-observation, fostering continuous growth and development.

### 2.4 Statistical methods

Data analysis was conducted using DMSAS 1.3.0 software. In Study 1, descriptive statistics, Pearson correlation analysis, and multiple regression analysis were performed. After controlling for demographic variables, mediation effect analysis was conducted to reveal the mediating role of psychological flexibility between social participation and retirement adaptation. In Study 2, paired sample t-tests were used to analyze and test the data before and after the intervention. This study was approved by the Ethics Committee of the Seventh People’s Hospital of Hangzhou.

## 3 Research results

### 3.1 Demographic characteristics analysis

The study sample mainly consisted of older adults aged 60 and above, with an average age of approximately 71 years (70.73 ± 6.67 years). The male-to-female ratio was 1:1. Regarding education level, 43.45% of participants had a college degree or higher, while 22.76% had no formal education or only an elementary school diploma, indicating a relatively high education level among the respondents. Regarding years of retirement, approximately 69.66% of participants had been retired for over 10 years. The sample distribution of job levels was relatively balanced. Concerning family situation, the majority of participants were married (84.14%) and maintained good relationships with their spouses. A small portion of the sample was unmarried, divorced, or widowed. Most participants’ spouses (97.24%) did not have a stable job or income, suggesting that they were also retired. Generally, participants reported having very good relationships with their children.

In terms of subjective economic status, most participants considered their economic condition to be good or average, with a small portion reporting it as very good, and only a few perceiving their economic condition as poor (5.52%).

### 3.2 Variable correlations

Table 3 shows the means, standard deviations, and correlation analysis of the variables. Social participation was positively correlated with retirement satisfaction (*r* = 0.25, *p <* 0.01) and positively correlated with nostalgia for work (*r* = 0.56, *p <* 0.001). These findings suggest that older adults who engage more in social activities tend to enjoy retirement more and experience more nostalgia for their previous work. Social participation was also positively correlated with retirement adaptation (*r* = 0.51, *p <* 0.001), indicating that higher levels of social participation were associated with better retirement adaptation.

**Table 3:** Means, standard deviations, and correlations of variables.

Variable	Means	Standard deviations	Social participation	Retirement satisfaction	Retirement loss	Nostalgia for work	Retirement adaptation	Psychological inflexibility
Social participation	9.77	4.19	1					
Retirement satisfaction	14.08	2.79	0.25**	1				
Retirement loss	10.81	4.03	−0.09	−0.49***	1			
Nostalgia for work	7.83	3.57	0.56***	0.01	−0.17*	1		
Retirement adaptation	32.71	4.58	0.51***	0.19*	0.45***	0.63***	1	
Psychological inflexibility	17.47	8.94	−0.49***	−0.14	−0.10	−0.47***	−0.54***	1

*p < 0.05, **p < 0.01, ***p < 0.001.

The mean score for psychological flexibility was 17.47, with a standard deviation of 8.94, indicating substantial variation in psychological flexibility among the older adults in the sample. AAQ-II scores were negatively correlated with both nostalgia for work (*r* = −0.47, *p* < 0.001) and retirement adaptation (*r* = −0.54, *p* < 0.001), indicating that greater psychological flexibility (i.e., lower AAQ-II scores) is associated with lower nostalgia for work and better adjustment to retirement.

These correlational patterns provide the necessary preconditions for subsequent mediation analysis; however, correlation alone cannot establish mediation, which requires regression and Bootstrap testing.

### 3.3 Regression analysis

After standardizing the data and controlling for demographic variables, linear regression analysis was conducted to examine the relationship between social participation and retirement adaptation among older adults. The results are presented in Table 4. According to the regression analysis in Table 4, after controlling for demographic variables such as gender, education level, years of retirement, relationship with children, job or technical level, marital status, spouse’s employment status, relationship with spouse, number of children, and relationship with children, social participation was found to have a statistically significant effect on retirement adaptation (*F* = 3.237, *p <* 0.001), explaining 30% of the variance (adjusted *R^2^* = 0.295). The change in model *R^2^* (Δ*R^2^* = 0.427) indicates that social participation plays an important role in predicting retirement adaptation.

**Table 4:** Regression analysis of social participation and retirement adaptation.

Variable	*B*	*SE*	*t*
Constant	27.45	5.36	5.12***
Gender	−1.69	0.79	−2.14*
Education level	−2.39	1.67	−1.44
Years of retirement	2.49	2.22	1.12
Job or technical level	0.76	1.07	0.71
Marital status	0.63	1.17	0.54
Spouse’s stable job or income	0.70	2.14	0.33
Relationship with spouse	−1.00	4.09	−0.24
Number of children	0.67	0.78	0.86
Relationship with children	0.30	1.61	0.19
*R^2^(abj)*		0.295	
△*R^2^*		0.427	
*F*		3.237***	

B = unstandardized regression coefficient; SE = standard error; t = t statistic. *p < 0.05, ***p < 0.001.

In terms of specific variables, gender exhibited a significant negative effect on retirement adaptation (*B =* -1.69, *t =* −2.14, *p <* 0.05), suggesting that women tend to have better retirement adaptation compared to men. The regression coefficient for education level was negative (*B =* -2.39, *t =* −1.44), indicating that higher educational levels were associated with lower retirement adaptation. Years of retirement were positively correlated with retirement adaptation (*B =* 2.49, *t =* 1.12), suggesting that the longer the retirement period, the better the adaptation. The regression coefficient for job or technical level prior to retirement was positive (*B =* 0.76, *t =* 0.71), indicating that older adults with higher technical or job levels tend to find it more difficult to adapt to retirement life.

Regarding marital status, the regression coefficient was positive (*B =* 0.63, *t =* 0.54), while the coefficient for relationship with spouse was negative (*B =* -1.00, *t =* −0.24), indicating that maintaining a good relationship with one’s spouse is beneficial for adapting to retirement life. The number of children was positively correlated with retirement adaptation (*B =* 0.67, *t =* −0.86), as was the relationship with children (*B =* 0.30, *t =* −0.19), suggesting that more children and better relationships with them contribute to better retirement adaptation.

Overall, social participation had a significant positive effect on retirement adaptation, even after controlling for other demographic variables. These results support Hypothesis 1 (H1).

### 3.4 Mediation effect

**Figure 1:**
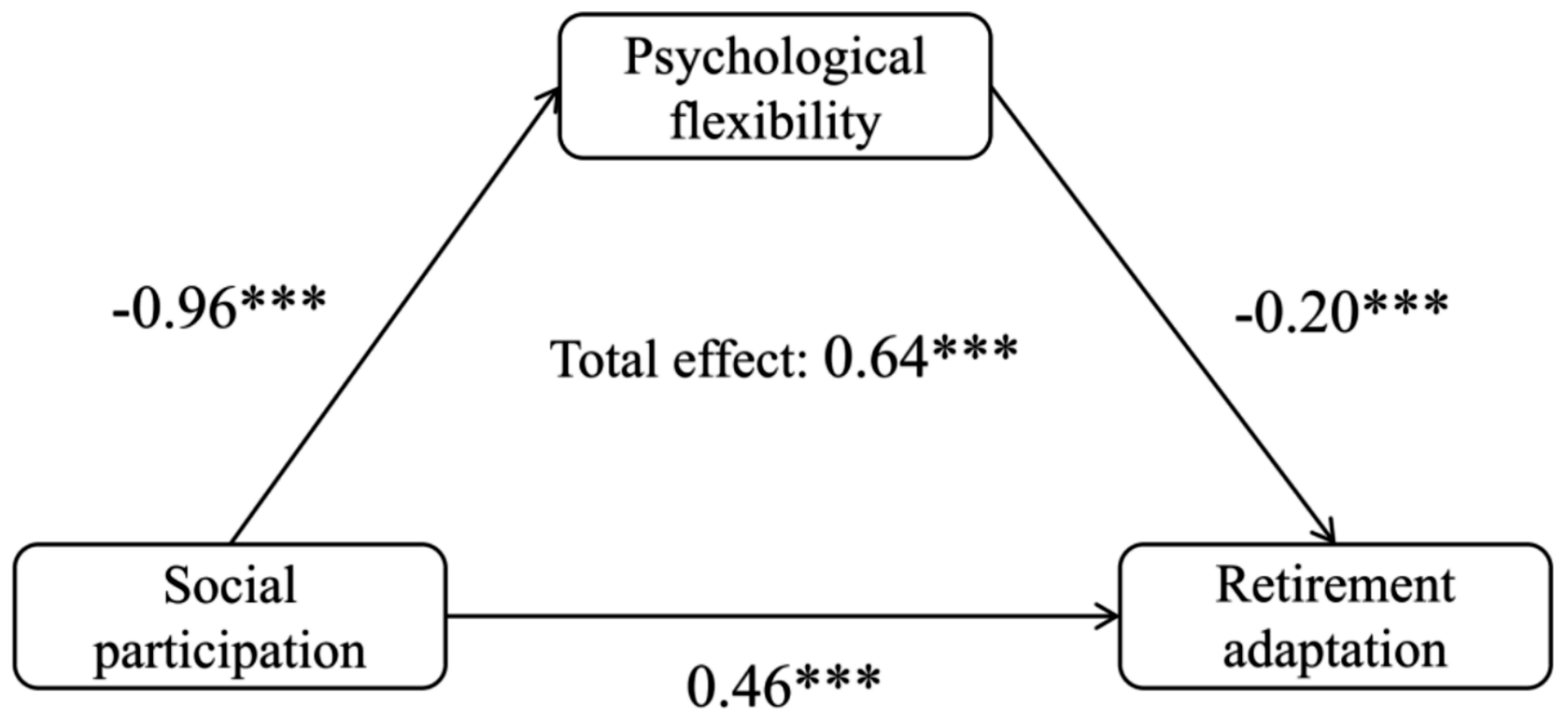
Path coefficients for the mediation effect of psychological flexibility. ***p < 0.001.

A mediation model was used with DMSAS 1.3.0 data analysis software, where social participation was the independent variable, retirement adaptation was the dependent variable, and psychological flexibility was the mediator. The path coefficients are shown in Figure 1.

The results indicate that social participation exerts a significant positive direct effect on retirement adaptation (*β* = 0.46, *p <* 0.001). Furthermore, in the mediation model using psychological inflexibility (AAQ-II scores) as the mediator: social participation significantly negatively predicted inflexibility (*β* = −0.95, *p* < 0.001), indicating that higher social participation was associated with lower inflexibility (i.e., higher psychological flexibility). Psychological inflexibility significantly negatively predicted retirement adaptation (*β* = −0.20, *p* < 0.001), indicating that lower inflexibility (higher flexibility) predicted better adaptation. Additionally, social participation has a direct effect on retirement adaptation, with a path coefficient of 0.46. The total mediation effect of psychological flexibility on retirement adaptation is 0.64. These results support H2.

Regression analysis (Table 5) indicated that social participation was a significant negative predictor of psychological flexibility (rigidity) (*β* = −0.96, *t* = −5.05, *p <* 0.01), with the model demonstrating a robust fit (*R =* 0.31, *R^2^* = 0.15, *F* = 1.92, *p <* 0.05). This suggests that increased social participation is associated with enhanced psychological flexibility, accounting for 15% of its variance. Furthermore, psychological flexibility significantly predicted retirement adaptation (*β* = −0.29, *t* = −7.42, *p <* 0.01), explaining 29% of the variance (*R =* 0.43, *R^2^* = 0.29, *F* = 3.22, *p <* 0.01), which reinforces the finding that higher psychological rigidity correlates with poorer retirement adjustment. When both social participation and psychological flexibility were entered into the integrated model, they collectively predicted retirement adaptation (*R =* 0.54, *R^2^* = 0.42, *F* = 4.79, *p <* 0.01). Specifically, social participation exerted a significant positive effect (*β* = 0.46, *t* = 5.25, *p <* 0.01), while psychological flexibility maintained a significant negative effect on adaptation (*β* = −0.20, *t* = −5.23, *p <* 0.01). These results indicate that psychological flexibility serves as a partial mediator in the relationship between social participation and retirement adaptation, thereby supporting Hypothesis H2.

**Table 5:** Regression analysis between variables.

Regression equation		Overall fit index			Regression coefficient significance	
Outcome variable	Predictor variable	*R*	*R^2^*	*F*	*B*	*t*
Psychological flexibility	Social participation	0.31	0.15	1.92**	−0.96	−5.05***
Retirement adaptation	Social participation	0.54	0.42	4.79***	0.46	5.25***
	Psychological flexibility				−0.20	−5.23***
Retirement adaptation	Psychological flexibility	0.43	0.29	3.22***	−0.29	−7.42***

R = correlation coefficient; R^2^ = coefficient of determination; F = F statistic; B = unstandardized regression coefficient; SE = standard error; t = t statistic. **p < 0.05, ***p < 0.001.

As presented in Table 6, the mediation analysis confirms the significant role of psychological flexibility in the relationship between social participation and retirement adaptation. The indirect effect was 0.189 (Boot SE = 0.056, 95% CI [0.098, 0.298]). Since the 95% confidence interval did not cross zero, it indicates that psychological flexibility significantly mediates the impact of social participation on retirement adjustment. The direct effect remained significant at 0.447 (Boot SE = 0.088, 95% CI [0.406, 0.610]), representing the direct contribution of social participation to adaptation levels. Consequently, the total effect was calculated at 0.636 (Boot SE = 0.085, 95% CI [0.460, 0.795]). The mediation proportion (29.73%) suggests that psychological flexibility serves as a partial mediator, bridging the path between social participation and successful retirement transition.

**Table 6:** Mediation effect of psychological flexibility between social participation and retirement adaptation.

Effect	Effect size	Boot SE	95% CI
Indirect effect	0.189***	0.056	[0.098, 0.298]
Direct effect	0.447***	0.088	[0.406, 0.610]
Total effect	0.636***	0.085	[0.460, 0.795]

SE = standard error, CI = confidence interval. **p < 0.01, ***p < 0.001.

### 3.5 Intervention results

A total of 37 participants were included in the control group and 40 in the intervention group. There were no significant differences in baseline characteristics between the two groups (*p >* 0.05). Paired sample t-tests were conducted to analyze the data. The pre- and post-intervention results for psychological flexibility and retirement adaptation are presented in Table 7, while the results for the three dimensions of retirement adaptation are shown in Table 8.

**Table 7:** Pre- and post-intervention comparisons of psychological flexibility and retirement adaptation.

Group	*n*	Psychological inflexibility		Retirement adaptation	
		Pre	Post	Pre	Post
Intervention group	40	14.75 ± 3.49	9.25 ± 2.24	32.20 ± 3.08	39.14 ± 11.33
Control group	37	14.59 ± 4.43	13.22 ± 2.29	32.30 ± 3.28	39.44 ± 10.56
*t*		0.417	−7.315	−0.459	1.379
*p*		0.678	<0.001	0.648	0.172

Values are presented as mean ± standard deviation. t = t statistic; p = *p*-value.

**Table 8:** Pre- and post-intervention comparisons of three dimensions of retirement adaptation.

Group	*n*	Retirement satisfaction		Retirement loss		Nostalgia for work	
		Pre	Post	Pre	Post	Pre	Post
Intervention group	40	14.93 ± 2.15	21.95 ± 2.41	7.53 ± 2.30	7.30 ± 2.59	9.75 ± 2.87	12.83 ± 2.34
Control group	37	14.89 ± 1.66	18.97 ± 2.10	7.68 ± 1.49	15.73 ± 2.33	9.73 ± 2.17	6.86 ± 3.13
*t*		−0.317	6.209	−0.120	−13.721	−0.220	9.532
*p*		0.753	<0.001	0.905	<0.001	0.827	<0.001

Values are presented as mean ± standard deviation. t = t statistic; p = *p*-value.

The results showed that after 6 weeks of intervention, both the control group and the intervention group exhibited significant improvements in retirement adaptation, indicating enhanced adaptation to retirement life. However, there was no significant difference between the two groups in terms of changes in retirement adaptation, suggesting that both general health education and ACT intervention can promote retirement adaptation among older adults, with comparable overall effects. These findings indicate that the interventions are beneficial in improving retirement adaptation and further support the effectiveness of ACT in enhancing psychological flexibility. Therefore, Hypothesis 3 (H3) is supported.

No significant change in psychological flexibility was observed in the control group before and after the intervention, whereas the intervention group showed a significant decrease in psychological inflexibility (*t =* −7.315, *p <* 0.001), indicating that ACT effectively improves psychological flexibility in older adults. Further analysis showed that, after the intervention, the intervention group demonstrated significant increases in retirement satisfaction (*t =* 6.209, *p <* 0.001) and nostalgia for work (*t =* 9.532, *p <* 0.001), while the control group exhibited a significant increase in retirement loss (*t =* −13.721, *p <* 0.001). These findings suggest that the mechanisms by which the control group and the intervention group influence retirement adaptation differ. Specifically, the control group tended to increase feelings of retirement loss, whereas the intervention group enhanced nostalgia for work and positive adaptation experiences.

## 4 Discussion

Based on the results of Study 1, social participation was found to have a positive effect on retirement adaptation, consistent with prior evidence from large-scale cohorts (21, 22). Its explanatory power (approximately 30%) aligns with the active aging framework’s view of social participation as a core behavioral pathway for functional maintenance and social integration (14, 22). However, the remaining 70% of unexplained variance suggests the presence of additional underlying mechanisms, which may include social networks, economic security, or psychological factors (23, 24). Furthermore, this study found that psychological flexibility plays a partial mediating role between social participation and retirement adaptation, supporting the role of psychological resources as a bridge between behavioral factors and adaptation outcomes, and aligning with the dynamic psychological resource model within the active aging framework (25).

Based on the results of Study 2, both groups showed improvements in retirement adaptation; however, the underlying mechanisms differed. The control group primarily exhibited an increase in retirement loss. This finding highlights the limitations of traditional health education. Although informational education can enhance cognitive reserves and covers a wide range of content, it may also increase anxiety related to retirement and intensify rumination processes among older adults (26). For example, while educating older adults about fall prevention may improve safety awareness, it may simultaneously heighten their perception of aging and anxiety about physical decline and falls. This finding is consistent with Cassanet et al. (12), who argued that purely information-based interventions may exacerbate cognitive and emotional dysregulation during the retirement transition period. Therefore, future health education interventions should shift from an “information-oriented” model to an integrated approach combining emotional support and psychological adjustment. For instance, incorporating digital health tools or virtual intervention platforms may further enhance the overall effectiveness of interventions (27).

In contrast, the intervention group showed significant improvements in both retirement satisfaction and nostalgia for work. Within the psychological flexibility hexaflex model, core processes such as acceptance, present-moment awareness, and self-as-context facilitate individuals in reducing experiential avoidance and overreactivity toward negative internal experiences, while enhancing openness to present-moment experiences (28). Accordingly, these processes may help older adults more fully engage with their current life situation after retirement, strengthen positive perceptions of daily experiences, and increase life satisfaction, thereby improving retirement adaptation, particularly in terms of retirement satisfaction.

In addition, although the intervention process may evoke recollections of past life experiences and a sense of contrast between pre- and post-retirement life, leading to potential retirement loss, ACT, unlike traditional health education, enables individuals to adopt a more flexible and non-judgmental stance toward internal experiences through cognitive defusion, values clarification, and committed action. This process allows older adults to reconnect post-retirement life with their core values. In doing so, they may reduce fixation on “role loss” and redirect attention toward meaningful and value-consistent actions, thereby facilitating a transition from “role loss” to “self-realization.” This mechanism is consistent with the active aging framework, which emphasizes meaning reconstruction and sustained social engagement, and also aligns with the dual ABC-X model, in which individuals adapt to stressors through resource reappraisal (14, 29, 30). Furthermore, this study found that although nostalgia for work significantly increased in the intervention group, it was not accompanied by a decline in overall retirement adaptation; instead, overall adaptation improved concurrently. This finding suggests that reflection on past work experience does not necessarily represent a negative outcome in older adults. Rather, it may reflect a process of integrative reminiscence. This process emphasizes the positive and meaning-making integration of past experiences, thereby enhancing self-continuity and life meaning, ultimately contributing to psychological well-being and adaptation in later life (31). Thus, the increased nostalgia observed here should be understood as an indicator of adaptive identity reconstruction rather than pathological role fixation.

Taken together, the findings from Study 1 and Study 2 demonstrate a strong complementary relationship across theoretical construction, methodological validation, and practical application, jointly elucidating the role and modifiability of psychological flexibility in retirement adaptation.

(1) Theoretical advancement: Study 1 identified psychological flexibility as a mediator between social participation and retirement adaptation, establishing an integrated “behavior (social participation) – psychological resource (psychological flexibility) – developmental outcome (retirement adaptation)” model, which provides a theoretical foundation for the intervention study. Study 2 further demonstrated through ACT-based intervention that psychological flexibility is a modifiable active component rather than a stable personality trait (32), suggesting that enhancing psychological flexibility may disrupt the maladaptive cycle linking social participation and retirement adjustment difficulties.

(2) From correlation to causality: While the cross-sectional design of Study 1 cannot establish temporal ordering, Study 2, employing a randomized controlled trial, provided preliminary evidence for the causal pathway in which ACT enhances retirement adaptation by increasing psychological flexibility (32). This approach is consistent with MacKinnon’s three-step framework for mediation testing, which involves identifying mediators and modifying them through intervention to observe changes in outcomes (33). However, given the short intervention duration (6 weeks) and the relatively small sample size (*n* = 77), the findings are insufficient to confirm a stable causal effect.

(3) Practical implications: These findings support the integration of psychological interventions into community-based older adults health management systems. At the community level, ACT, mindfulness training, and values-based interventions may be incorporated to improve adaptation capacity and quality of life among older adults, thereby promoting the goals of active aging (14).

## 5 Limitations and future directions

Several limitations should be acknowledged in this study. First, the data were primarily collected using self-report questionnaires, which may introduce a degree of subjective bias. In particular, older adult participants may require assistance from researchers when completing the questionnaires, which may further increase social desirability bias. This may lead participants to report overly positive responses. Therefore, future studies should consider employing multiple data collection methods, such as behavioral observations and physiological indicators, to further validate questionnaire-based findings.

Second, this study may be subject to sample selection bias. The participants were primarily recruited from Hangzhou, Zhejiang Province, China, where educational attainment and socioeconomic status are relatively high. This may limit the generalizability of the findings to populations with different socioeconomic backgrounds.

In terms of the intervention design, due to time and resource constraints, only a single control group was included. To improve the robustness and applicability of the findings, future research should consider incorporating placebo or additional control groups. A three-arm or multi-arm design would allow for more precise comparisons of intervention effects across different conditions, thereby enhancing external validity and providing more refined practical guidance.

In addition, changes in psychological flexibility may be dynamic and non-linear. Early fluctuations in intervention scores may reflect the loosening of defensive psychological mechanisms. Therefore, future studies should adopt longitudinal follow-up designs to further examine its long-term trajectories and its relationship with adaptation outcomes (34).

Despite these limitations, the present study provides important evidence for understanding the psychological mechanisms and intervention pathways of retirement adaptation. Future research should further explore potential moderating variables (such as social support and personality traits) and validate the proposed model across different cultural contexts, in order to develop more generalizable and culturally sensitive intervention strategies for aging populations.

## 6 Conclusion

This study systematically examined the role and modifiability of psychological flexibility in the relationship between social participation and retirement adaptation through a combination of cross-sectional analysis and randomized controlled intervention design. The results of Study 1 demonstrated that psychological flexibility plays a significant partial mediating role between social participation and retirement adaptation, revealing an underlying pathway through which social behavioral factors influence adaptation outcomes via psychological resources. This suggests that older adults who actively engage in social activities may enhance their psychological flexibility, thereby facilitating a smoother transition into retirement. Social participation provides greater social and emotional support, strengthening older adults’ ability to cope with retirement-related challenges, while higher psychological flexibility enables more effective emotion regulation, lifestyle adjustment, and coping with life transitions after retirement.

Study 2 provided preliminary experimental support for the role of psychological flexibility in retirement adaptation. Acceptance and Commitment Therapy (ACT) significantly improved psychological flexibility and concurrently enhanced retirement adaptation, indicating that psychological flexibility is not only a correlational variable but also a modifiable and key intervention target. These findings are consistent with the hypothesis that ACT may promote retirement adaptation by enhancing psychological flexibility, but the specific mechanisms underlying this association require further empirical investigation.

Taken together, these findings outline a “social participation–psychological flexibility–retirement adaptation” pathway that integrates correlational and experimental evidence. However, firm conclusions regarding the causal mediation chain await replication in larger samples with longer follow-up periods.

From a practical perspective, this study highlights psychological flexibility as a key entry point for mental health interventions in older adults, suggesting a shift from single-component health education toward a multidimensional intervention model combining behavioral promotion and psychological intervention. Specifically, combining opportunities for social participation (e.g., community activities and volunteer services) with evidence-based interventions such as ACT may help older adults reconstruct meaning in life and enhance adaptive capacity. In addition, targeted psychological support should be provided for individuals with higher occupational status or greater role transition difficulties to reduce their risk of poor retirement adaptation.

Overall, this study not only enriches the theoretical understanding of the psychological mechanisms underlying retirement adaptation but also provides important evidence for developing intervention strategies aimed at promoting health, participation, and well-being in older adults. These findings are highly consistent with the core principles of active aging and healthy aging frameworks, which emphasize functional maintenance and enhancement of psychological resources.

## Data Availability

The original contributions presented in the study are included in the article/supplementary material, further inquiries can be directed to the corresponding author.
